# Weapon use during the index offense: a study among forensic psychiatry patients in Ontario, Canada

**DOI:** 10.1186/s40621-024-00551-z

**Published:** 2024-12-18

**Authors:** Mark Mohan Kaggwa, Arianna Davids, Parwiz Mohibi, Bailea Erb, John Bradford, Gary Andrew Chaimowitz, Andrew Toyin Olagunju

**Affiliations:** 1 Department of Psychiatry and Behavioral Sciences, McMaster University, St Joseph’s Healthcare Hamilton, 100 West 5th Hamilton, Ontario, L89 3K7 Canada; 2 Forensic Psychiatry Program, St Joseph’s Healthcare Hamilton, 100 West 5th Hamilton, L89 3K7 Ontario, Canada; 3 Department of Psychiatry, Mbarara University of Science and Technology, Mbarara, Uganda; 4 Michael G DeGroote School of Medicine, McMaster University, Hamilton, Ontario, Canada; 5 Department of Psychology, University of Waterloo, Ontario, Canada; 6 Division of Forensic Psychiatry, University of Ottawa, Ontario, Canada; 7 Department of Psychiatry and Behavioral Sciences, University of Oklahoma College of Medicine, Oklahoma City, OK 73117 USA; 8 Discipline of Psychiatry, The University of Adelaide, Adelaide, SA 5005 Australia

**Keywords:** Forensic psychiatry, Index offense, Ontario, Risk, Victim relationship, Weapons use

## Abstract

**Background:**

Understanding the nature and circumstances around the use of weapons to perpetrate an offense among individuals with mental illness is crucial for evidence-informed policies and actions. However, little is known about the prevalence and factors associated with weapon use during index offenses among patients in the forensic system. Therefore, the present study was designed to address this gap and determine the prevalence and the patient and victim characteristics associated with weapon use during the index offense in a Canadian province.

**Methods:**

This retrospective exploratory study utilized data extracted from the Ontario Review Board reports of 2014/15. Data was analyzed using Stata, and logistic regression was employed to determine the factors associated with weapon use.

**Results:**

Approximately half (48.11%) of the individuals included in this analysis (*n* = 819) used weapons during their index offense as an instrument of violence. Both patient-related and victim-related factors had a statistically significant association with weapon use during index offenses. Specifically, two patient-related factors (including a history of hospitalization prior to the index offense and diagnosis of personality disorder) were associated with lower odds of weapon use during the index offense. However, only prior hospitalization remained statistically significant after adjusting for victims’ factors. Victim-related factors were associated with both lower and higher odds of weapon use during the index offense. The highest odds of weapon use were found if the victim was an extended family member of the patient, followed by sibling, lover/partner/spouse, parent, and then adult acquaintance. The odds of weapon use during the index offense were lower if victims were healthcare workers, law enforcement professionals, and females when compared to adult strangers.

**Conclusion:**

The study highlights the significant role of both patients’ and victims’ characteristics as important factors associated with weapon use during index offenses among forensic patients. Notably, prior hospitalization emerged as a crucial factor with a reduced likelihood of weapon use. Implicitly, this underscores the importance of risk mitigation strategies.

## Introduction

In Canada, individuals with severe mental illness who come into contact with the law may be found Not Criminally Responsible (NCR) or Unfit to Stand Trial (UST) for their committed crimes, known as index offenses [1, 2]. Individuals with NCR or UST status are then detained in the forensic mental health system to undergo court or tribunal-ordered psychiatric treatment to enhance recovery, risk mitigation and community re-integration as forensic psychiatric patients [1, 2]. A significant portion of the index offenses associated with forensic patients are violent in nature, comprising 69.9% of cases in the Canadian province of Ontario from 2014 to 2015 [3]. However, the nature of the violence varies widely, ranging from verbal threats and physical assaults to the use of offensive weapons [2, 3].

One critical aspect when examining violent crimes is to ascertain whether the individual used offensive weapons (e.g., firearms) during their index offense. The National Trajectory Project (NTP), which examined 1800 cases of individuals found NCR, found that the use of offensive weapons was relatively low among forensic psychiatric patients, with a prevalence rate of 9.9% in British Columbia, 7.9% in Ontario, and 4.6% in Quebec [4]. Despite these lower rates, examining and understanding the circumstances around weapon use in the context of violent crime is crucial because the use of weapons during an assault is more likely to cause significant physical harm to the victim, including harm that may be lethal compared to assaults without weapons [5]. Additionally, higher rates of recidivism are found among individuals who use weapons during the index offense compared to those who do not [6]. Offenders who use weapons are also more likely to reoffend using weapons [7]. Therefore, understanding weapon use and its associated factors is critical in predicting and preventing future weapon use among forensic psychiatric patients.

The literature on weapon use during an index offense by forensic psychiatric patients is limited. However, studies on other groups, such as juvenile offenders, have identified several factors linked to weapon use. Behavioral and social factors such as substance use, poor impulse control, gang membership, and having peers who use guns can significantly influence violent behavior [8, 9]. Psychiatric and psychological factors, including psychopathy and delusional disorders, also play a role [10, 11]. For instance, mothers experiencing psychosis are more likely to use weapons in filicide, while younger children are less likely to be killed with weapons in such cases [10]. Gender-specific factors reveal that women often use kitchen knives, and higher blood alcohol levels are linked to women using sharp weapons [8, 12]. Demographic factors show that fathers are usually killed with firearms, while mothers are more often killed with cutting weapons or physical assault [11]. Minors use firearms more frequently than adults, and both adults and youths tend to kill their fathers with firearms [11]. Forensic psychiatric factors indicate that the type of mental disorder can influence weapon choice, with conditions like delirium or hallucinations leading to the use of unusual weapons, such as fire [11]. While various factors may influence the likelihood of weapon use among offenders, the specific factors and their relationships are largely under explored in forensic psychiatric research. This paper aims to fill this gap by examining the prevalence of offensive weapon use during an index offense and providing better insight into plausible factors that may explain weapon use in forensic settings.

This study examines patients’ characteristics, such as age, gender, psychiatric diagnosis, and hospitalization history, as well as victim characteristics, including the victim relationship, age, and gender, and how these factors relate to weapon use during the index offense. Understanding the relationships of these factors with weapon use can inform the development of evidence-guided risk mitigation strategies for forensic psychiatry patients. Furthermore, by understanding the interplay between patient characteristics, victim characteristics, and the use of weapons during a violent index offense, it becomes possible to create effective and risk-tailored preventative interventions, policies, and action plans.

## Methods

The current exploratory study utilizes retrospective data collected from reports submitted to the Ontario Review Board (A provincial Tribunal Court that oversees patients’ progress within the forensic system in Ontario) during the reporting year of 2014 to 2015 (*N* = 1240). Some of the data from this database have been previously utilized and described in other publications [3, 13, 14]. This study was approved by the Hamilton Integrated Research Ethics Board (HiREB); the approval number is #15,564.

### Study variables

Only individuals with data that captured weapon use (the main outcome explored in this study) were included in the analysis conducted for the present study. The covariables examined included patient characteristics such as age at the time of the index offense(s), gender, previous hospitalization for a psychiatric condition, primary psychiatric diagnosis, and intoxication at the time of the offense. Similarly, victim characteristics, including the victim’s gender, age, and their relationship with the patient were also considered.

### Analysis plan

Descriptive statistics were presented using frequencies and percentages for categorical variables. Mean and standard deviation was used to present the continuous variable (age of patient during the index offense) that was normally distributed, as assessed by visual inspection of the histogram. The age of victims, which was not normally distributed, was described using the median and interquartile range. The distribution of the victim’s age was assessed using visual inspection of the box and whisker plot and the histogram. The difference between weapon users and non-users was tested using a two-sample Wilcoxon test.

Bivariate and multivariate logistic regression analyses were used to determine the factors associated with weapon use to answer the main research question: what factors were associated with weapon use during the index offense among patients admitted to a forensic psychiatric hospital? The main outcome variable was weapon use, which was dichotomized (yes or no). The predictor variables were gender, age, psychiatric diagnosis, previous hospitalization, victim’s gender, victim’s age, and victim’s relationship to the patient. The bivariate analysis tested the association between each predictor variable and the outcome variable, and the results were presented as crude odds ratios (cOR) with 95% confidence intervals (CI) and p-values. A p-value of less than 0.05 was considered statistically significant. The null hypothesis for each test was that there was no association between the predictor and outcome variable, and the alternative hypothesis was that there was an association. The predictor variables that were significant during the bivariate analysis and the biologically plausible factors were included in the multivariate model. The factors were tested for collinearity (using Variance Inflation Factor [VIF]) before being included in the multivariate model. Despite the victim’s age having no issues with collinearity, many of the victims’ ages were missing, and to avoid further power reduction, it was not included in the final model. After controlling for the confounding effects of the other variables, the multivariate analysis tested the adjusted association between the predictor variables and the outcome variable. The results were presented as adjusted odds ratios (aOR) with 95% CI and p-values. The null hypothesis for the multivariate model was that there was no independent association between the predictor variables and the outcome variable, and the alternative hypothesis was that there was at least one predictor variable independently associated with the outcome variable. The model fit and the goodness of fit were assessed using the Hosmer-Lemeshow test and the Nagelkerke R-squared.

## Results

### Participants characteristics

Out of the 819 forensic psychiatric patients included in this analysis, 48.11% (*n* = 394) used weapons during the index offense as an instrument of violence. The average age of the patients was 34.27 (standard deviation of 12.20) years at the time of the index offense, and most of the patients identified as male (*n* = 701, 85.59%). The victims of violence were predominantly male (*n* = 394, 48.11%), and most of them were strangers to the patients (*n* = 254, 31.01%) (Table 1**)**.

**Table 1:** Distribution of patients and victims’ characteristics among forensic patients with weapon use at their index offense

Variable	Details	Descriptive statistics *N* = 819	Weapons			
			Null hypothesis	No, *n* (%) 425 (51.89)	Yes, *n* (%) 394 (48.11)	X^2^/t/W (*p*-value)
**Patients’ characteristics**						
Age (years)	Age at the index offense	Mean 34.27 (Standard Deviation = 12.20)	The mean age of weapon use is equal to that who did not use weapons	34.89 (11.67)	33.61 (12.73)	1.50 (0.134)
Gender (as reported by patient)	Male	701 (85.59)	Weapon use during the index offence and gender are independent	361 (51.50)	340 (48.50)	0.30 (0,582)
	Female	126 (14.53)		64 (54.24)	54 (45.76)	
Previous hospitalization for a psychiatric condition	No	121 (14.85)	Weapon use during the index offence and Previous hospitalization for a psychiatric condition are independent	41 (33.88)	80 (66.12)	**18.48 (< 0.001)**
	Yes	694 (85.15)		382 (55.04)	312 (44.96)	
Primary psychiatric diagnosis	Psychotic disorder	680 (83.03)	Weapon use during the index offence and primary psychiatric diagnosis are independent	345 (50.74)	335 (49.26)	**11.63 (0.020)**
	Mood disorder	52 (6.35)		33 (63.46)	19 (36.54)	
	Neurodevelopmental disorder	25 (3.05)		11 (44.00)	14 (56.00)	
	Personality disorder	29 (3.54)		22 (75.86)	7 (24.14)	
	Others	33 (4.03)		14 (42.42)	19 (57.58)	
Intoxication at the time of the index offense	No	710 (86.69)	Weapon use during the index offence and intoxication at index offence are independent	373 (52.54)	337 (47.46)	7.72 (0.172)
	Drugs	41 (5.01)		16 (39.02)	25 (60.98)	
	Alcohol	42 (5.13)		24 (57.14)	18 (42.86)	
	Yes (not specified)	5 (0.61)		1 (20.00)	4 (80.00)	
	Both (alcohol and drugs)	7 (0.85)		2 (28.57)	5 (71.43)	
	Unknown	14 (1.71)		9 (64.29)	5 (35.71)	
**Victims’ characteristics**						
Victim gender	Male	394 (48.11)	Weapon use during the index offence and victim’s gender are independent	179 (45.43)	215 (54.57)	**20.11 (< 0.001)**
	Female	358 (43.71)		197 (55.03)	161 (44.97)	
	Unknown	67 (8.18)		49 (73.13)	18 (26.87)	
Victim age	Age in years at the time of the offence (717 victims had the age missing from the records)	Median = 33.5 and Interquartile range = 12–61	The median age between those who used weapon during the index offence use and those who did not are equal	15 (8–54)	49 (18–64)	**1751.5 (0.014)**
Victims’ relationship with the patient	Stanger – adult	236 (28.82)	Weapon use during the index offence and victim’s relationship are independent	132 (55.93)	104 (44.07)	**96.75 (< 0.001)**
	Stranger – child	18 (2.20)		13 (72.22)	5 (27.78)	
	Acquaintance – adult	115 (14.04)		45 (39.13)	70 (60.87)	
	Acquaintance – child	3 (0.37)		2 (66.67)	1 (33.33)	
	Friend	16 (1.95)		6 (37.50)	10 (62.50)	
	Parent	113 (13.80)		39 (34.51)	74 (65.49)	
	Son/daughter	20 (2.44)		12 (60.00)	8 (40.00)	
	Sibling	31 (3.79)		10 (32.26)	21 (67.74)	
	Lover/partner/spouse	43 (5.25)		15 (34.88)	28 (65.12)	
	Other family members	24 (2.93)		4 (16.67)	20 (83.33)	
	Law enforcement professionals	83 (10.13)		61 (73.49)	22 (26.51)	
	Healthcare/support staff	66 (8.06)		55 (83.33)	11 (16.67)	
	Co-habitant/co-patient	23 (2.81)		13 (56.52)	10 (43.48)	
	Others	10 (1.22)		4 (40.00)	6 (60.00)	
	Unknown	18 (2.20)		14 (77.78)	4 (22.22)	

The age of the victims was not normally distributed; it was skewed to the right, and most of the victims were below the age of 20 years. The median age of the victims was 33.5, and the interquartile range was between 12 and 61 years. No outliers in the sample related to age existed, but 717 victims had no age recorded/captured.

### Relationship of weapon use with patients and victims’ characteristics during index offense

The results from the chi-square test of independence showed there were significant associations between weapon use during the index offense and the following variables: (i) previous hospitalization for a psychiatric condition (χ^2^ = 18.48, *p*-value < 0.001), (ii) primary psychiatric diagnosis (χ^2^ = 11.63, *p*-value = 0.020), victim’s gender (χ^2^ = 20.11, *p*-value < 0.001), and victim’s relationship to the patient (χ^2^ = 96.75, *p*-value < 0.001). These results indicate that the null hypothesis of no association between weapon use and each of these variables can be rejected at the 0.05 significance level. Among those who had used weapons during the index offense, the proportion of those who had previous hospitalization for a psychiatric condition was significantly lower (44.96% vs. 66.12%, *p*-value < 0.001) (See Table 1 for details). A two-sample Wilcoxon test was performed to test whether the median age of victims differed between those attacked with and without weapons at the time of the index offense. The results showed that the median age of victims who were attacked with a weapon was significantly higher than the median age of victims who were not assaulted with a weapon (49 vs. 15, W = 1751.5, *p*-value = 0.014). The mean age of patients who used a weapon at the time of the index offense was similar to the mean age of patients who did not use a weapon (34.89 vs. 33.61). The p-value of the student’s t-test was above 0.05 (*t* = 1.50, *p*-value = 0.134), meaning that we did not have enough evidence to reject the null hypothesis that the means were equal (Table 1).

### Patient factors associated with weapon use during the index offense

At bivariate analysis, the following patients’ characteristics were associated with lower odds of weapon use at the time of the index offense: (i) having previous hospitalization due to a psychiatric illness (cOR = 0.42, 95% CI [0.28–0.63]; p-value < 0.001) and (ii) having a diagnosis of a personality disorder (cOR = 0.33, 95% CI [0.14–0.78]). In other words, the odds of using a weapon at the time of the index offense were 58% lower for those with previous psychiatric hospitalization than for those without. Since the p-value was less than 0.05, we can reject the null hypothesis of no association between the predictor and outcome variables at the 0.05 significance level. However, after adjusting for other factors in a multivariate model, including victim characteristics (except victim age), having previous hospitalization due to a psychiatric condition had 57% lower odds of using a weapon during the index offense than those who were never admitted, and this association was statistically significant (aOR = 0.43, 95% CI [0.27–0.67]; *p*-value < 0.001) (Table 2).

**Table 2:** Logistic regression analysis for factors associated with weapon use among forensic psychiatric patients in Ontario

Variables		Null hypothesis	Bivariate regression analysis		Multivariate regression analysis	
			Crude odds ratio (95% confidence interval)	*p*-value	Adjusted odds ratio (95% confidence interval)	*p*-value
**Patients’ characteristics**						
Age (years)		Age of the patients at the index offence has no effect on the log odds of having used a weapon during the index offence	0.99 (0.98–1,00	0.134	1.00 (0.99–1.01)	0.828
Gender (as reported by patient)	Male	There is no linear relationship between the gender of patients and log odds of having used a weapon during the index offence	1		1	
	Female		0.90 (0.61–1.32)	0.582	1.07 (0.69–1.67)	0.752
Previous hospitalization for a psychiatric condition	No	There is no linear relationship between having history of being hospitalized prior to index offence and log odds of having used a weapon during the index offence	1		1	
	Yes		0.42 (0.28–0.63)	**< 0.001**	0.43 (0.27–0.67)	**< 0.001**
Primary psychiatry diagnosis	Psychotic disorder	There is no linear relationship between the primary diagnosis and log odds of having used a weapon during the index offence	1		1	
	Mood disorder		0.59 (0.33–1.06)	0.079	0.65 (0.34–1.24)	0.190
	Neurodevelopmental disorder		1.31 (0.59–2.93)	0.509	1.71 (0.67–4.36)	0.258
	Personality disorder		0.33 (0.14–0.78)	**0.011**	0.43 (0.16–1.16)	0.097
	Others		1.40 (0.69–2.83)	0.353	1.89 (0.85–4.22)	0.118
Intoxication at the time of the index	No	There is no linear relationship between being intoxicated during the index offence and log odds of having used a weapon during the index offence	1		1	
	Drugs		1.73 (0.91–3.29)	0.096	1.92 (0.93–3.94)	0.076
	Alcohol		0.83 (0.44–1.56)	0.562	0.58 (0.29–1.18)	0.136
	Yes (not specified)		4.43 (0.49–39.80)	0.184	2.22 (0.22–21.98)	0.495
	Both (alcohol and drugs)		2.77 (0.53–14.36)	0.226	1.98 (0.34–11.34)	0.443
	Unknown		0.61 (0.20–1.85)	0.388	0.55 (0.17–1.78)	0.319
**Victim characteristics**						
Victim gender	Male	There is no linear relationship between the victim’s gender and log odds of having used a weapon during the index offence	1		1	
	Female		0.68 (0.51–0.91)	**0.009**	0.54 (0.38–0.76)	**< 0.001**
	Unknown		0.31 (0.17–0.54)	**< 0.001**	0.58 (0.30–1.12)	0.107
Victim age	Age in years at the time of the offence	Victim’s age of the patients at the index offence has no effect on the log odds of having used a weapon during the index offence	1.02 (1.00–1.03)	**0.027**		
Victim relationship	Stranger – adult	Victim’s relation to the patient has no effect on the log odds of having used a weapon during the index offence	1		1	
	Stranger – child		0.49 (0.17–1.41)	0.186	0.44 (0.14–1.34)	0.149
	Acquaintance – adult		1.97 (1.25–3.11)	**0.003**	1.70 (1.06–2.74)	**0.029**
	Acquaintance – child		0.63 (0.56–7.09)	0.712	0.38 (0.03–4.98)	0.461
	Friend		2.11 (0.74–6.01)	0.160	1.18 (0.38–3.66)	0.768
	Parent		2.41 (1.51–3.83)	**< 0.001**	2.64 (1.60–4.31)	**< 0.001**
	Son/daughter		0.85 (0.33–2.15)	0.725	0.69 (0.25–1.92)	0.479
	Sibling		2.66 (1.20–5.91)	**0.016**	3.14 (1.37–7.16)	**0.007**
	Lover/partner/spouse		2.37 (1.20–5.91)	**0.013**	2.74 (1.32–5.60)	**0.007**
	Other family members		6.35 (2.10–19.14)	**0.001**	6.61 (2.13–20.54)	**0.001**
	Law enforcement professionals		0.46 (0.26–0.79)	0.005	0.39 (0.21–0.71)	**0.002**
	Healthcare/support staff		0.25 (0.13–0.51)	**< 0.001**	0.27 (0.13–0.55)	**< 0.001**
	Co-habitant/co-patient		0.98 (0.41–2.31)	0.957	0.82 (0.34–2.02)	0.672
	Others		1.90 (0.52–6.92)	0.328	1.54 (0.41–5.80)	0.526
	Unknown		0.36 (0.12–1.13)	0.081	0.50 (0.14–1.70)	0.265

**Model statistics** All the variables included in the final model had a VIF of less than 2 and a mean VIF of 1.05. In addition, the final model had a goodness of fit chi-square value of 657.29 and a p-value of 0.227, meaning that we did not have enough evidence to reject the null hypothesis that the model had a good fit and that the probability of having a chi-square value this high or higher was above 0.05

### Victim’s characteristics associated with weapon use during the index offense

All victim-related factors were significantly associated with the patients’ weapon use in the bivariate logistic regression analysis. In the multivariate logistic regression model, after adjusting for other factors, the following victim factors were significantly associated with increased odds of being attacked with a weapon, with the highest odds associated with being an extended family member of the patient (aOR = 6.61, 95% CI [2.13–20.54], *p*-value < 0.001) followed by sibling (aOR = 3.14, 95% CI [1.37–7.16], *p*-value = 0.007), then lover/partner/spouse (aOR = 2.74, 95% CI [1.32–5.60], *p*-value = 0.007), then parent (aOR = 2.64, 95% CI [1.60–4.31]; *p*-value < 0.001), and least odds were associated with being an adult acquaintance (aOR = 1.70, 95% CI [1.06–2.74]; *p*-value = 0.029) (Table 2). This means that the odds of being attacked by a weapon were 6.61 times higher for victims who were extended family members of the patient compared to victims who were adult strangers. The 95% confidence interval of the odds ratio did not include one (i.e. Above one), indicating a statistically significant and positive association between the patients’ weapon use and being their extended family member. The *p*-value was less than 0.05, meaning there was a very low probability of observing such a large or larger odds ratio by chance alone if the null hypothesis of no association were true.

At multivariate regression, the odds of being attacked by a weapon were 0.27, 0.39, and 0.54 times the odds of not being attacked by a weapon for victims who were healthcare workers, law enforcement officers, and females, respectively, compared to adult strangers. The 95% confidence interval of the odds ratios did not include 1 and was below 1, indicating a statistically significant and negative association between the aforementioned characteristics and outcome variable. The *p*-value was less than 0.05, which meant that there was a very low probability of observing such an odds ratio by chance alone if the null hypothesis of no association were true.

## Discussion

This study examined the factors associated with weapon use among patients admitted to forensic psychiatric hospitals in Ontario during their index offense. Roughly half (48.11%) of the patients included in this analysis (*n* = 394) used weapons during the index offense as an instrument of violence. Both patient-related factors and victim-related factors had a statistically significant association with weapon use during the index offense. Specifically, two patient-related factors (including psychiatric hospitalization prior to the index offense and a diagnosis of personality disorder) were associated with lower odds of weapon use during the index offense. However, only prior hospitalization remained statistically significant after adjusting for victim factors. Victim-related factors were associated with both lower and higher odds of weapon use during the index offense. The highest odds of weapon use were found if the victim was an extended family member of the patient, followed by sibling, lover/partner/spouse, parent, and then adult acquaintance. The odds of weapon use during the index offense were lower when victims were healthcare workers, law enforcement professionals, and females when compared to adult strangers.

The prevalence of weapon use in the present study (48.11%) was notably higher than the 7.9% reported in the National Trajectory Project (NTP) for Ontario, which included 484 forensic patients in Ontario [4]. This discrepancy may be attributed to the differences in sample selection. The NTP focused exclusively on individuals with severe index offenses who were found Not Criminally Responsible (NCR) between 2000 and 2005 [4, 15]. In contrast, our study encompassed all groups of patients in the Ontario forensic system during the 2014 and 2015 reporting year, thus including a broader and more recent cohort. Specifically, our study included both NCR patients and those deemed Unfit to Stand Trial (UST), unlike the cohort in the NTP, which only considered individuals with NCR status. This inclusion of UST patients, who may have different risk profiles, could further explain the elevated prevalence of weapon use in our findings. Moreover, the higher prevalence observed in the present study may reflect recent shift in the characteristics of the patients (i.e., beyond 2005) in the forensic system due to improved data on weapon use and an increasing trend in weapon use over time [16].

On a different note, the prevalence of weapon use during an index offense in our study (48.11%) is slightly lower than findings reported by de Vogel and de Spa [17]. In their study, over half of the forensic psychiatric patients (56.9% of women and 57.3% of men) used a weapon during their index offense. Several factors may contribute to this discrepancy. Firstly, the difference in sample size (*N* = 249 from one facility in the Netherlands versus *N* = 819 from several facilities in a province in Canada) could play a role. The population in one facility in the Netherlands may include higher-profile patients (individuals who may pose a higher risk) compared to a more general representation of forensic patients in the province of Ontario. Higher-profile patients are usually managed in higher secure facilities and may have a history of previously being involved in severe offenses that involved the use of weapons. Another important consideration for the discrepancy in the prevalence rates is the potential variations in the operational definitions of what constitutes a weapon. For instance, de Vogel and de Spa [17] included medication/poison as a definition for a weapon; however, the definition of a weapon during the index offense in the present study was codified to match the definition outlined in Sect. 2 of the Canadian Criminal Code [18] - which defines a “weapon” as any item used, designed to be used, or intended for use in causing death or injury to any person or for the purpose of threatening or intimidating any person. While this definition broadly includes firearms, knives, and other objects that can cause harm or instill fear, it does not include items such as medicines and poisons. The differences in the definitions of a weapon highlight the complexity of categorizing weapons and underscore the need to use standardized criteria in forensic psychiatric research for large data, transnational or meta-analytic analysis.

While our study found that a diagnosis of a personality disorder was associated with lower odds of weapon use during an index offense, few studies have also examined this relationship, albeit not specifically among forensic patients. One Finnish study by Saukkonen and colleagues [2016] found that psychopathic-like features were related to a higher likelihood of weapon carrying by adolescents, even after adjusting for other risk factors [19]. However, it must be noted that this study examined adolescents and did not focus on forensic psychiatric patients; therefore, the difference in weapon use associated with personality disorders should be interpreted with caution. Another 2020 study by Robertson and colleagues [2021]found that juvenile offenders with callous-unemotional traits—which are often linked to psychopathy—had an increased probability of gun use during a crime [20]. Again, this study looked at juveniles, did not consider personality disorders directly, and was not conducted in a forensic psychiatric system. Swanson and colleagues [2015], in their study of a sample in the United States, found that persons with anger traits who carried guns were significantly more likely to meet the diagnostic criteria for personality disorders (as well as other mental health disorders), also showing a higher association between personality disorders and weapon use compared to the findings in our study [21]. This may be because this population was looking at the United States (where carrying firearms is legal), people who carried guns (as opposed to other weapons), were not limited to offending using the weapon and did not examine a forensic population. Comparisons in this regard are difficult to draw as there is limited literature on weapon use by forensic psychiatric patients with personality disorders. It is also important to note that the findings in the current study results used patients with psychotic disorders (who are often associated with persecutory beliefs or paranoia that may drive the use of weapons in attempts to protect themselves) as a reference. This may be a rare occurrence in cases of individuals with personality disorders where malice or desire to cause harm to others may be the motivator, thus the lesser likelihood. Also, the classification of personality disorder was open, thus not allowing for proper attribution to a particular type of personality disorder that may be associated with weapon use. Due to these limitations, we hope the results are interpreted cautiously, and the findings are carefully generalized to all forensic patients diagnosed with a personality disorder.

Similarly, the authors could not find any studies that specifically examined the association between prior psychiatric hospitalization and weapon use during an index offense, thereby making direct comparisons to other literature challenging. This gap in the literature underscores the need for further research on this specific association. Existing literature suggests that psychiatric treatment and effective control of symptoms are protective against violence [22]. For instance, a study by Elbogen and colleagues found that psychiatric community treatment significantly reduces the risk of violent behavior among individuals with severe mental illness [22]. This aligns with our hypothesis that individuals with prior hospitalization had a mitigated risk of using weapons to perpetuate violence and were at a lower risk of causing significant harm to others. Also, prior hospitalization likely indicates that these individuals received more intensive treatment and monitoring, which could contribute to better symptom management and potential reduction in impulsivity or aggression. Moreover, psychiatric hospitalization often involves comprehensive risk assessments and the development of personalized treatment plans, which can address underlying issues contributing to violent behavior, such as the use of weapons. Also, prior psychiatric hospitalization may lead to interventions such as weapons removal, thus explaining reoffending, albeit with less weapons use Overall, our study finding that prior hospitalization is associated with lower odds of weapon use among forensic patients highlights the crucial role of mental health treatment in mitigating the risk of violence. In this regard, access to psychiatric care, maintenance treatment with ongoing support, monitoring, and follow-up care for individuals with severe mental illness are important. Future studies to better understand this association could provide valuable insights for improving risk assessment and management strategies in forensic psychiatric systems, and ultimately contribute to better patient outcomes and enhanced public safety.

We found that the odds of being attacked with a weapon are highest for extended family members of forensic patients, followed by siblings, lovers/partners/spouses, parents, and lastly, adult acquaintances. This suggests that closer familial relationships are associated with higher odds of being victims of violence perpetrated with a weapon during an offense. Several factors may contribute to this pattern, including conflicts within families, especially extended families, which can be more emotionally charged and intense, leading to a greater likelihood of weapon use during disputes. The dynamics of trust and betrayal are often more pronounced in familial relationships, and when trust is broken, the emotional response can be more severe, potentially leading to violent outcomes involving weapons [23]. Additionally, many individuals with mental illness may be homeless or transient, moving between family members, including extended family, which increases their access to these relatives and opens up opportunities for harm [24]. Family members, especially those living in the same household or frequently visiting, have more opportunities for interaction, increasing the chances of conflicts escalating to violence that may involve weapons. Despite families being victims when weapons are used, there was no association found when the family member was a son or a daughter. This lack of association may highlight the enduring strength of parental love and care, even in situations involving mental illness. It suggests that the protective instincts and deep emotional bonds parents have with their children might mitigate the likelihood of weapon use in these relationships, underscoring the unique dynamics of parental relationships compared to other familial connections. Another potential explanation for this observed finding could be that resorting to weapon use may be rare when attacking a young child or smaller individual [10].

The findings also indicate that people in authoritative positions (e.g., healthcare workers and law enforcement professionals) have significantly lower odds of being attacked with a weapon compared to adult strangers. Healthcare workers are often viewed as caregivers and helpers, which may reduce the likelihood of them being targeted with weapons. Additionally, healthcare settings typically have strict security measures and protocols to protect staff from violence. This is also positively impacted by existing measures and advocacies for the prevention of violence towards health workers from patients with mental illness [25]. The professional relationship and care context may also contribute to lowering aggression levels towards healthcare workers. Law-enforcement professionals, such as police officers and other personnel, are trained to handle violent situations and are often equipped with protective gear and weapons themselves. This training and equipment can act as deterrents to potential attackers. Moreover, the legal consequences of attacking a law enforcement officer are severe, which may further discourage such attacks. In Canada, for instance, assaulting a public or peace officer can result in imprisonment for up to five years [26].

Our study also showed a reduced likelihood of female victims being attacked with a weapon. This could be influenced by societal norms and perceptions. Attackers may perceive women as less threatening, reducing the likelihood of using a weapon.

### Limitations

Interpretation of these study results should be in light of the following limitations. Firstly, the specific weapons used were unknown, and the term “weapon” can be broad, including knives, firearms, and blunt objects, which may affect the associations observed. Secondly, missing data reduces the power of the analysis. Additionally, many variables were divided into very small categories, such as the exact familial relationship between the victim and perpetrator, making it difficult to identify overarching associations. Lastly, as a retrospective study, causality cannot be inferred.

## Conclusion

The use of weapons to perpetrate violence during their index offense is highly prevalent among individuals in the forensic psychiatric system. This is a major issue for risk mitigation and management. The victims’ relationship with the patients, patients’ gender, and their occupation significantly influenced the likelihood of weapon use by patients during their index offense. Similarly, previous encounters with psychiatric care appear to decrease the odds of perpetrating violence with weapons. Based on these study results, community management of psychiatric patients is key in reducing the likelihood of using weapons during the index offense. Furthermore, there is a need for policies, action plans and interventions that are informed by current knowledge of the explanatory factors for weapon use among forensic patients to mitigate the risk.

## Data Availability

No datasets were generated or analysed during the current study.

## References

[1] Askola R, Soininen P, Seppänen A. Offense-related issues in forensic Psychiatric treatment: a thematic analysis. Front Psychiatry. 2019;10:925. 31998150 10.3389/fpsyt.2019.00925 PMC6961555

[2] Latimer J. Les systèmes de commissions d’examen au Canada: Review board systems in Canada: overview of the results from the mentally disordered accused data collection study. 2007.

[3] Chaimowitz G, Moulden H, Upfold C, Mullally K, Mamak M. The Ontario Forensic Mental Health System: a Population-based review. Can J Psychiatry. 2022;67(6):481–9. 34109832 10.1177/07067437211023103 PMC9152242

[4] Crocker AG, Nicholls TL, Seto MC, Charette Y, Côté G, Caulet M. The National Trajectory Project of individuals found not criminally responsible on Account of Mental Disorder in Canada. Part 2: the people behind the label. Can J Psychiatry. 2015;60(3):106–16. 25886686 10.1177/070674371506000305 PMC4394710

[5] Leesti T. Weapons and violent crime. Volume 17. Citeseer; 1997.

[6] Hans Z, Cooper CE, Zeoli AM. Examining the role of firearm involvement in repeat intimate partner violence assaults. Inj Epidemiol. 2024;11(1):9. 38439114 10.1186/s40621-024-00492-7 PMC10910667

[7] Rydberg J, McGarrell E, De Biasi F. Firearms offending and re-offending. In: Michigan Justice statistics Center. Michigan State University School of Criminal Justice; 2023. https://cj.msu.edu/_assets/pdfs/mjsc/MISAC_Report_FirearmsArresteesStudy_Dec2023.pdf. Accessed 5 Sept 2024.

[8] Lerer LB. Women, homicide and alcohol in cape town, South Africa. Forensic Sci Int. 1992;55(1):93–9. 1511943 10.1016/0379-0738(92)90098-h

[9] Pardini D, Beardslee J, Docherty M, Schubert C, Mulvey E. Risk and protective factors for Gun Violence in male juvenile offenders. J Clin Child Adolesc Psychol. 2021;50(3):337–52. 33124922 10.1080/15374416.2020.1823848 PMC8925316

[10] Lewis CF, Baranoski MV, Buchanan JA, Benedek EP. Factors Associated with Weapon Use in maternal Filicide. J Forensic Sci. 1998;43(3):613–8. 9608698

[11] Catanesi R, Carabellese F, Troccoli G, Candelli C, Grattagliano I, Solarino B, Fortunato F. Psychopathology and weapon choice: a study of 103 perpetrators of homicide or attempted homicide. Forensic Sci Int. 2011;209(1):149–53. 21316880 10.1016/j.forsciint.2011.01.019

[12] Rogde S, Hougen HP, Poulsen K. Homicide by sharp force in two scandinavian capitals. Forensic Sci Int. 2000;109(2):135–45. 10704816 10.1016/s0379-0738(99)00230-3

[13] Kaggwa MM, Chaimowitz GA, Erb B, Prat S, Davids A, Moulden H, Robbins A, Bradford J, Mamak M, Olagunju AT. Self-harming behaviors and forensic system-related factors: an analysis of the Ontario review board database. BMC Psychiatry. 2023;23(1):913. 38057757 10.1186/s12888-023-05394-4 PMC10698976

[14] Kaggwa MM, Chaimowitz GA, Erb B, Moulden H, Prat S, Davids A, Olagunju AT. Adverse childhood events and self-harming behaviours among individuals in Ontario forensic system: the mediating role of psychopathy. BMC Psychiatry. 2024;24(1):332. 38693475 10.1186/s12888-024-05771-7 PMC11064378

[15] Crocker AG, Charette Y, Seto MC, Nicholls TL, Côté G, Caulet M. The national trajectory project of individuals found not criminally responsible on account of mental disorder in Canada. Part 3: trajectories and outcomes through the forensic system. Can J Psychiatry. 2015;60(3):117–26. 25886687 10.1177/070674371506000306 PMC4394711

[16] Allen M. Trends in firearm-related violent crime in Canada, 2009 to 2020. In: *Juristat*. Volume 42. Statistics Canada; 2022.

[17] de Vogel V, de Spa E. Gender differences in violent offending: results from a multicentre comparison study in Dutch forensic psychiatry. Psychol Crime Law. 2019;25(7):739–51.

[18] Branch L. Consolidated federal laws of canada, criminal code. In: *RSC, 1985, c C-46.* Edited by Law Ja, vol. R.S.C., 1985, c. C-46. Canada: Justice and Law website; 2022.

[19] Saukkonen S, Laajasalo T, Jokela M, Kivivuori J, Salmi V, Aronen ET. Weapon carrying and psychopathic-like features in a population-based sample of Finnish adolescents. Eur Child Adolesc Psychiatry. 2016;25(2):183–91. 25986501 10.1007/s00787-015-0724-2

[20] Robertson EL, Frick PJ, Ray JV, Thornton LC, Wall Myers TD, Steinberg L, Cauffman E. Do callous–unemotional traits moderate the effects of the juvenile justice system on later offending behavior? J Child Psychol Psychiatry. 2021;62(2):212–22. 32449527 10.1111/jcpp.13266

[21] Swanson JW, Sampson NA, Petukhova MV, Zaslavsky AM, Appelbaum PS, Swartz MS, Kessler RC. Guns, impulsive angry behavior, and Mental disorders: results from the National Comorbidity Survey replication (NCS-R). Behav Sci Law. 2015;33(2–3):199–212. 25850688 10.1002/bsl.2172 PMC5116908

[22] Elbogen EB, Van Dorn RA, Swanson JW, Swartz MS, Monahan J. Treatment engagement and violence risk in mental disorders. Br J Psychiatry. 2006;189(4):354–60. 17012659 10.1192/bjp.bp.105.017913

[23] Holst ME. Understanding our extended families: Predictors and outcomes. *M.S.* United States -- Iowa: Iowa State University; 2014.

[24] Nijdam-Jones A, Nicholls TL, Crocker AG, Roy L, Somers JM. History of Forensic Mental Health Service Use among homeless adults with Mental Illness. Int J Forensic Mental Health. 2017;16(1):69–82.

[25] World Health Organization. Preventing violence against health workers. *Available from*: https://www.whoint/activities/preventing-violence-against-health-workers 2022

[26] Government of Canada. Justice Laws Website. Criminal Code RSC. 1985, c C-46, s 270 [https://laws-lois.justice.gc.ca/eng/acts/c-46/section-270.html]

